# Spotted Lanternfly Feeding Induces Systemic Defense Responses in Grapevines

**DOI:** 10.1007/s10886-026-01742-2

**Published:** 2026-07-24

**Authors:** Sukhman Singh, Michelle Peiffer, Gary W. Felton, Flor E. Acevedo

**Affiliations:** Department of Entomology, The Pennsylvania State University, PA 16802 University Park, USA

**Keywords:** *Lycorma delicatula*, Plant hopper, *Vitis vinifera*, Plant defenses, Sap feeding insects

## Abstract

The spotted lanternfly (*Lycorma delicatula*), an invasive planthopper in the United States, threatens vineyards by reducing yield and compromising plant health, yet grapevine immune responses to its feeding remain poorly understood. This study evaluated systemic grapevine responses to spotted lanternfly feeding under varying insect densities and feeding durations. Potted *Vitis vinifera* cultivar Cabernet Franc vines were infested with 0, 5, 10, or 15 adults per vine for 1, 3, 5, 7, 14, 21, or 29 days, using separate plants at each time point. Leaf tissue was analyzed for chlorophyll content, polyphenol oxidase (PPO) and peroxidase (POX) activity, protein content, total phenolics, and condensed tannins. Bioassays assessed spotted lanternfly survival and weight gain on previously infested plants. Chlorophyll content declined after 21–29 days in vines infested with 15 insects. PPO activity increased throughout the experiment (except on day 7) under infestations of 10–15 insects, whereas POX activity rose after 7 days but declined after 29 days of feeding. Leaf protein content increased in vines infested with 15 insects after 7 and 29 days, and phenolic concentrations increased consistently from 7 to 29 days in vines infested with 10–15 insects. Condensed tannin concentrations were unaffected by density treatments. Bioassays showed reduced survival and weight gain of lanternflies when fed on vines previously infested with 15 insects for 29 days. Collectively, these results suggest that prolonged, high-density spotted lanternfly feeding induces coordinated, time- and density-dependent systemic grapevine defenses associated with reduced host suitability. Future studies should identify the mechanisms underlying these responses and their persistence across grapevine cultivars.

**Supplementary Information:**

The online version contains supplementary material available at https://doi.org/10.1007/s10886-026-01742-2.

## Introduction

The spotted lanternfly, *Lycorma delicatula* White 1845 (Hemiptera: Fulgoridae), is a phloem-feeding insect native to Southeast Asia that has become an invasive pest in the United States since its detection in 2014. This insect feeds on the phloem sap of a wide range of host plants, but it is particularly damaging to grapevines, posing a significant threat to the grape and wine industry (Huron et al. 2022). Previous studies have linked heavy spotted lanternfly feeding to reductions in grapevine photosynthesis, stomatal conductance, leaf nitrogen content, non-structural carbohydrates in stems and roots, root biomass, crop yield, and fruit quality (Harner et al. 2022, 2025). Despite these documented physiological effects, it remains unclear whether grapevines activate systemic defenses that modify plant quality and influence subsequent herbivore performance.

Plants have evolved sophisticated defense mechanisms that enable them to perceive herbivore attack and reduce subsequent damage (Howe and Jander 2008; Karban 2011; War et al. 2012, 2018). These defenses may be constitutively present or induced in response to insect herbivory (Gatehouse 2002; Wu and Baldwin 2009; Hu et al. 2024). Herbivore feeding induces early signaling events, including the generation of reactive oxygen species (ROS) and calcium fluxes (Kanchiswamy et al. 2010; Zebelo and Maffei 2015; Gandhi et al. 2021; Gao et al. 2022), which activate downstream pathways mediated by phytohormones such as jasmonic acid (JA), salicylic acid (SA), and ethylene (ET), among others (Bari and Jones 2009; Erb et al. 2012). These signaling pathways stimulate the production of defense-related enzymes and secondary metabolites that reduce tissue palatability, interfere with insect digestion, and reduce herbivore growth and survival (Felton et al. 1992; Felton 2005; War et al. 2020).

Among the induced chemical defenses, polyphenol oxidases (PPOs), peroxidases (POXs), and phenolic compounds play central roles. PPOs oxidize phenolics into reactive o-quinones, which reduce the nutritional quality of plant tissue and damage insect gut epithelial cells. Oxidative quinones can also reduce the digestibility of plant tissue, diminishing insect growth and survival. POXs oxidize phenolic compounds, decreasing digestibility and impairing insect growth and development (Felton et al. 1992; Felton 2005). POXs also regulate plant ROS homeostasis, contribute to cell wall strengthening, and promote recovery from oxidative stress (Gechev et al. 2006; Dey et al. 2020; Dalio et al. 2021; Torres 2010; Sharma et al. 2012; Do et al. 2003; Schaffer and Bronnikova 2012). Herbivory often induces the accumulation of phenolic compounds and other secondary metabolites that further decrease host suitability for insects (War et al. 2020; Singh et al. 2021). For example, infestation by the grain aphid *Sitobion avenae* (Hemiptera: Aphididae) increases phenolic concentrations in winter triticale, resulting in enhanced plant resistance to aphids (Chrzanowski and Leszczyński 2008). Collectively, these defense enzymes and compounds can alter plant quality, deter insect feeding, limit herbivore performance, and influence insect behavior.

Evidence that grapevines activate defense responses upon spotted lanternfly feeding has been documented previously. Islam et al. (2022) investigated transcriptional responses of grapevines (6-year-old hybrid Marquette) to heavy and prolonged spotted lanternfly infestation (80 adults per vine for 45 days), focusing on localized changes in metabolic and defense-related pathways. Their findings showed that several genes involved in the SA and JA signaling pathways were upregulated in response to spotted lanternfly feeding. Additionally, genes associated with the biosynthesis of secondary metabolites, particularly phenolics, and genes encoding peroxidases, which contribute to cell wall lignification, were enriched (Islam et al. 2022). Interestingly, genes linked to photosynthesis were also upregulated. Their study provided valuable insights into grapevine responses to spotted lanternfly feeding, but our understanding of grapevine systemic responses to varying insect densities and feeding durations and their effects on subsequent herbivore performance remains limited.

Plant responses to herbivory develop progressively, with early signaling events activating downstream metabolic pathways that lead to physiological and biochemical changes, including the accumulation of defense enzymes and secondary metabolites (Karban 2011; Kant et al. 2015; Ladeynova et al. 2023). The timing, magnitude, and persistence of these responses vary among plant-herbivore systems and are influenced by herbivore feeding mode, infestation intensity, and feeding duration (Karban 2011; Kant et al. 2015). Despite growing recognition that sap-feeding insects elicit plant responses distinct from those induced by chewing insects, little is known about how grapevines respond over time following spotted lanternfly feeding.

In this study, we investigated how spotted lanternfly density and feeding duration influence systemic defense responses in grapevines and whether these responses affect subsequent spotted lanternfly performance. We hypothesized that (1) spotted lanternfly feeding activates systemic responses in grapevines that increase with infestation density and feeding duration, and (2) these induced responses decrease host suitability, resulting in reduced spotted lanternfly fitness. To test these hypotheses, we quantified physiological responses (chlorophyll content), activation of defense-related enzymes (PPO and POX), accumulation of secondary metabolites (total phenolics and condensed tannins), and leaf protein content. We also conducted insect bioassays to determine whether these induced responses altered spotted lanternfly survival and weight gain. By integrating plant biochemical responses with herbivore performance, this study advances our understanding of grapevine-spotted lanternfly interactions.

## Materials and Methods

*Insect Colony.* For bioassays, insects were reared in a greenhouse from egg hatch to adult emergence. Egg masses were collected from the Penn State Fruit Research and Extension Center in Biglerville, PA, in mid-April 2024 and stored in a cooler at 8 ℃. Egg masses were removed from the cooler on three separate dates (May 19, June 2, and 18, 2024). On each date, ~ 75 egg masses were placed in pop-up cages and provided with tree of heaven and grapevines (*V. vinifera* cultivar Cabernet Franc) for feeding after hatching. For plant infestation experiments, we collected spotted lanternfly adults on August 5th, 2024, from Bluemont Vineyards, Bluemont, VA, and Huntington, PA. Adults were kept in mesh cages (90*60*60 cm, Jinhua Quiangsheng Outdoor Products, Zhejiang, China) and fed with tree of heaven and grapevines (*V. vinifera* cultivar Cabernet Franc) for about a week before being used for experiments.

*Plant Material.* Rooted grapevine canes (*V. vinifera* cultivar Cabernet Franc grafted onto 101 − 14 rootstock) were purchased from a certified nursery (Double A Vineyards, Fredonia, New York) in the Spring of 2024 and were stored at 4 ℃ until planting. The roots of each cane were trimmed to about 4 inches (10 cm) before being planted into 2.5-gallon pots filled with a 1:2 mixture of topsoil and growth media (Premier Pro-mix BX with Mycorrhizae, Premier Horticulture Inc.) on May 28th – 29th, 2024. Three weeks later, the plants were pruned to keep 3–4 shoots per vine. One month after planting, the vines were fertilized with 50 g of Osmocote Plus (N:15, P:9, K:12). The vines were grown in a Pennsylvania State University’s glasshouse facility (temperature: ~27/17 ℃ (day/night), relative humidity: 60% and natural photoperiod) at Mendel’s Way, Bellefonte, PA, and watered as needed throughout the experiment to avoid water limitation in all treatments. All vines were pruned to regulate growth; however, no pruning was conducted within 14 days before the start of the experiment at any time point to minimize potential pruning-induced wound responses and ensure the availability of new leaf growth for sample collection. Control vines were subjected to the same pruning method as infested plants.

*Experimental Setup.* To study grapevine systemic responses to spotted lanternfly feeding, we conducted a time course and insect density-dependent experiment. In mid-August 2024, we infested individual grapevines enclosed in mesh cages (90*60*60 cm) with either 0 (control), 5, 10, or 15 adult spotted lanternflies and collected leaf tissue for analyses at 1, 3, 5, 7, 14, 21, and 29 days post-infestation. The insect densities were chosen based on preliminary data and on a previous field study that reported a naturally occurring range of 3.2 to 60 spotted lanternfly adults per vine (Leach and Leach 2020). Each density and time point had 10 replications, and a different set of plants was used at each time point. The spotted lanternfly numbers in each cage were checked every day, and the densities were maintained by replacing dead insects with those kept in a greenhouse colony. Newly fully expanded leaves from 2 to 3 shoots of each vine were collected at each time point, flash-frozen in liquid nitrogen, and stored at -80 ℃ for further analysis. Cages containing grapevines were arranged in a randomized complete block design with the greenhouse benches serving as the blocking factor and a two-factorial treatment structure consisting of time points and insect density. Within each block (bench), all time point combinations and density treatments were randomly assigned.

*Chlorophyll Content.* 100 mg of ground leaf tissue was homogenized in 1 mL 100% methanol and kept at room temperature for 1 h in the dark. Subsequently, the samples were centrifuged at 11,000 g for 5 min, and the supernatant was collected and stored in the dark. The extraction and centrifugation steps were repeated three times, and the resulting 3 mL supernatant was combined. 200 $\:\mu\:$L of the supernatant was used in a 96-well plate, and the absorbance was measured in a spectrophotometer (SpectraMax 190, Molecular Devices) at 665 and 652 nm for chlorophyll determination (Lichtenthaler 1987). The following formulas were used for calculating chlorophyll content:

- Chlorophyll a (µg/g tissue) = (((16.72 × A665) – (9.16 × A652)) * Volume in ml)/weight of sample in g.
- Chlorophyll b (µg/g tissue) = (((34.09 × A652) - (15.28 × A665)) * Volume in ml)/weight of sample in g.
- Total Chlorophyll ( µg/g tissue) = (((1.44 × A665) + (24.93 × A652)) * Volume in ml)/weight of sample in g.

*Enzyme Activity.* Peroxidase (POX) and polyphenol oxidase (PPO) activity in grapevine leaves was examined following the protocol described by Serradell et al. (2000), Chung and Felton (2011), and Acevedo et al. (2017), with some modifications. To examine the POX and PPO activity, 80 mg of leaf tissue was ground in a 2 mL tube using a Geno grinder. One mL of ice-cold acetone (100%) was added to each tube, and the tubes were placed on ice for 90 min. All samples were centrifuged at 11,000 g for 5 min, and the supernatant was discarded after centrifugation. A 200 $\:\mu\:$L solution of 5% cross-linked polyvinylpyrrolidone (PVPP), 0.1% Triton X-100, and 1 M sodium chloride in 0.1 M potassium phosphate buffer (pH 7.0) was added to each sample and kept on ice for 15 min. All samples were centrifuged at 11,000 g for 5 min. 10 $\:\mu\:$L of the supernatant, 10 $\:\mu\:$L of 3% hydrogen peroxide, and 200 $\:\mu\:$L of 0.25% guaiacol were added to a 96-well plate, and absorbance was measured at 450 nm to determine POX activity using a spectrophotometer. To measure PPO activity, 10 $\:\mu\:$L of the sample and 200 $\:\mu\:$L of 3mM caffeic acid were added to a 96-well plate, and absorbance was measured at 450 nm on a spectrophotometer. Readings were taken for 5 min at 9-second intervals and expressed as mOD min^− 1^ mg^− 1^ of fresh tissue.

*Leaf Protein Content.* We used a trichloroacetic acid-acetone method to determine protein content in grapevine leaves (Jellouli et al. 2010). 100 mg of leaf tissue was ground, and 400 $\:\mu\:$L of ice-cold acetone containing 8% (v/v) of tri-chloroacetic acid, 2% (w/v) of PVPP, and 2% (v/v) of $\:\beta\:$-mercaptoethanol was added. Proteins were precipitated overnight at -20 ℃ and centrifuged for 15 min at 10,000 g. The supernatant was discarded, and the pellet was resuspended in 400 $\:\mu\:$L of ice-cold acetone containing 0.1% $\:\beta\:$-mercaptoethanol and kept at -20 ℃ for 1 h. The samples were centrifuged for 15 min at 10,000 g, and the pellet was washed three times with 500 $\:\mu\:$L of acetone containing 0.1% $\:\beta\:$-mercaptoethanol. The supernatant was discarded, and the pellet was air-dried for 10 min, followed by the addition of 100 $\:\mu\:$L of 0.1 M Tris-HCl (pH 8.0) to solubilize the proteins for 15 min on ice. Protein content was determined with the Bradford method using bovine serum albumin as a standard.

*Total Phenolics.* The concentration of total phenolics was determined using the Folin-Ciocalteu (F-C) protocol (Ainsworth and Gillespie 2007). 1.5 mL of ice-cold 95% methanol was added to 30 mg of ground leaf tissue. The samples were vortexed and incubated for 48 h at room temperature in the dark, followed by centrifugation at 13,000 g for 5 min at room temperature. 100 $\:\mu\:$L of the supernatant was transferred to new tubes, and 200 $\:\mu\:$L of 10% F-C reagent was added to each sample. Subsequently, 800 $\:\mu\:$L of 700 mM sodium carbonate was added to each sample and incubated at room temperature for 2 h. Afterwards, 200 $\:\mu\:$L of the sample was transferred to a 96-well microplate, and the absorbance was read at 765 nm. A standard curve was generated using gallic acid to quantify total phenolics, which were expressed as gallic acid equivalents (GAE) per mg of fresh leaf tissue.

*Condensed Tannins.* 100 mg of ground leaf tissue was homogenized in 2 mL of 100% methanol and kept in the dark for 1 h. The samples were centrifuged at 11,000 g for 5 min, and the supernatant was recovered. An aliquot of 25 $\:\mu\:$L of the extract was mixed with 225 $\:\mu\:$L of 1% vanillin and 8% HCl (2:1, vanillin: HCl) and incubated at room temperature for 20 min. Absorbance was measured at 500 nm using a spectrophotometer, and a standard curve was generated using catechin. The concentration of condensed tannins was expressed as mg catechin equivalents per g of fresh leaf tissue (Broadhurst and Jones 1978).

*Bioassays.* We conducted bioassays to evaluate the effect of systemic grapevine responses induced by spotted lanternfly feeding on the survival and weight gain of recently emerged spotted lanternfly adults. After collecting leaf samples from plants exposed to 0, 10, and 15 adults for 7, 14, 21, and 29 days post-infestation, the plants were placed back in cages, and one pre-weighed, newly molted (within 24 h) spotted lanternfly adult was introduced into each cage. Mortality was recorded every other day, and the adults were weighed every seven days for four weeks to assess weight gain.

*Experimental Design and Statistical Analysis.* We used a factorial randomized complete block design with insect density and feeding duration as fixed factors and greenhouse benches as a blocking factor to account for variation in sun exposure. We used a two-way factorial analysis of variance (ANOVA) to determine the effects of insect density (Factor 1), time of feeding (Factor 2), and their interaction (density x time) on chlorophyll content, enzyme activity (PPO, POX), protein content, total phenolics, condensed tannins, and phytohormone concentrations. Differences between treatment means were analyzed with the Tukey-HSD test at alpha 0.05. In cases where the interaction term was significant, a one-way ANOVA was conducted to elucidate the effect of insect density at given feeding time points, and the *P*-values were adjusted for multiple testing using the Bonferroni correction; the raw values were multiplied by the number of tests conducted, and their significance was evaluated at alpha = 0.05. Data were tested for normal distribution, and appropriate transformations were applied when necessary. Spotted lanternfly mortality in the bioassays was analyzed using Kaplan-Meier survival curves, and weight gain over time was analyzed using a linear mixed-effects model (LMM) to account for repeated measurements on the same insect over time. Each pre-infestation timepoint (7, 14, 21, and 29) was analyzed separately because insects emerged at different times, and the plants exposed to the pre-infestation treatments became available at one-week intervals. Within each pre-infestation timepoint, weight gain was modeled as a function of time during the bioassay (6, 14, 22 and 29 days for the pre-infestation time points of 7 and 14 days; 7, 14, 21 and 28 for the 21-day pre-infestation time point; and 7 and 14 days for the 29-day pre-infestation time point), including insect densities (0, 10, and 15 adults per plant), and their interaction. Because one insect was placed on each plant and weighed repeatedly, plant (insect) identity was included as a random intercept to account for non-independence of repeated observations. Model assumptions were assessed using residual diagnostics, and when significant effects were detected, Tukey-adjusted pairwise comparisons of estimated marginal means were conducted. All analyses were carried out using the R software.

## Results

**Fig. 1:**
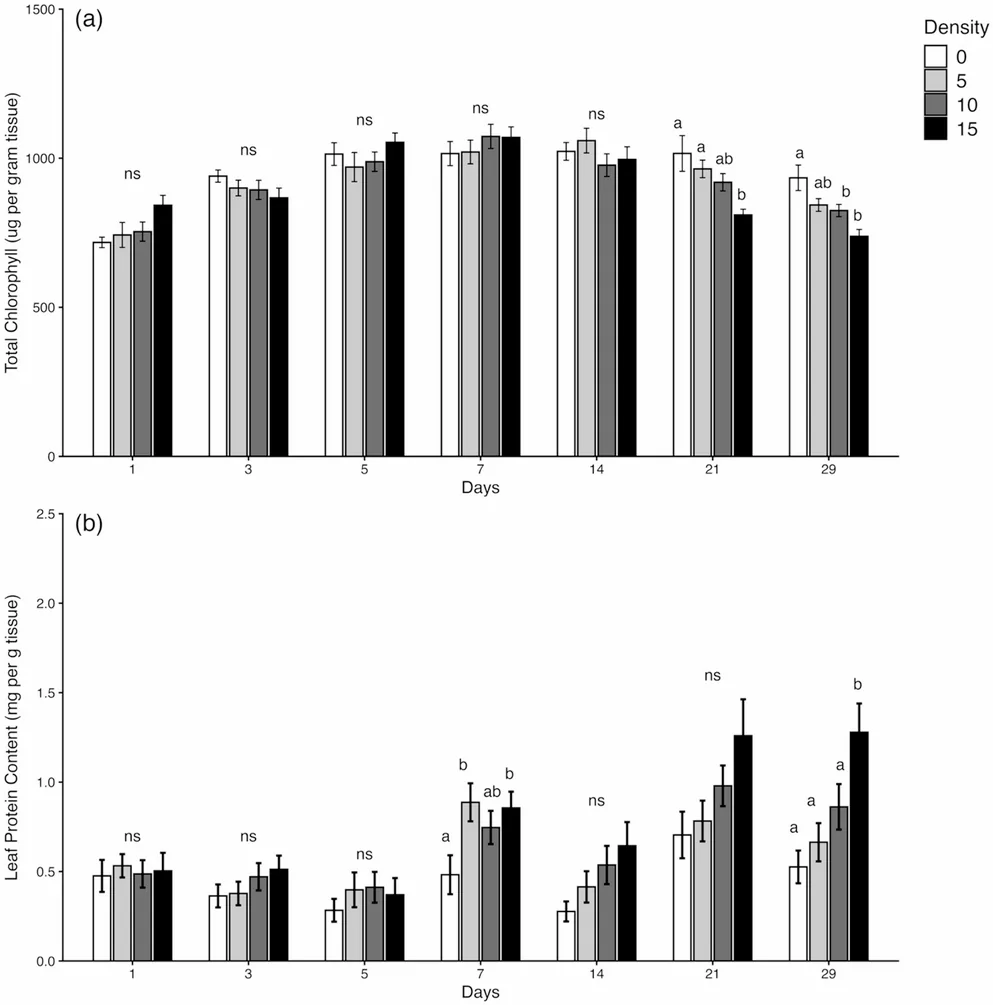
(**a**) Total chlorophyll and (**b**) leaf protein content in grapevine leaves exposed to 0, 5, 10, and 15 spotted lanternfly adults per plant for 1, 3, 5, 7, 14, 21, and 29 days. ANOVA. Letters on bars represent significant differences among treatments (Tukey test at *P* < 0.05) and ‘ns’ denotes non-significance (Tukey test at *P* > 0.05). Bars represent means $\:\pm\:$ standard error

*Spotted Lanternfly Reduced Leaf Chlorophyll Content After Three Weeks of Consecutive Feeding.* The total chlorophyll content varied significantly over time (*F* = 35.18, *P* < 0.0001) and was affected by the interaction between insect density and feeding duration (Density*Days: *F* = 2.69, *P* = 0.0003), but not by insect density alone (*F* = 1.84, *P* = 0.14). Because the interaction term for the two-way ANOVA was significant, separate one-way ANOVAs were conducted to determine the effect of insect densities at each time point. Significant differences were detected in total chlorophyll content among insect densities at 21 (ANOVA; *F* = 5.409, *P*-adj = 0.00479; Fig. 1) and 29 (ANOVA; *F* = 8.347, *P*-adj = 0.000435; Fig. 1a) days post-infestation, but not at earlier timepoints. Specifically, at day 21, grapevines exposed to 15 insects had lower total chlorophyll content when compared with those fed on by five insects or non-infested controls (Fig. 1a). After 29 days of feeding, grapevines exposed to 10 and 15 insects had lower chlorophyll content when compared with untreated controls (Fig. 1a). Measurements of chlorophyll A indicated no detectable effect of spotted lanternfly feeding (Supplementary Fig. 1a). In contrast, the chlorophyll B content was significantly reduced in grapevines exposed to 29 days of herbivory by 10 and 15 spotted lanternfly adults per plant compared with controls (Supplementary Fig. 1b).

*Spotted Lanternfly Infestation Increased Leaf Protein Content.* The protein content in grapevine leaves was significantly affected by the different insect densities (*F* = 12.556, *P* < 0.0001) across feeding times (*F* = 22.591, *P* < 0.0001), with a significant effect of their interaction (Density x Day: *F* = 1.908, *P* = 0.0168). One-way ANOVA revealed significant differences in leaf protein content among insect densities at days 7 (*F* = 6.344, *P*-adj = 0.0124) and 29 (*F* = 10.948, *P*-adj = 0.0005), but not at other time points. At day 7, the leaf protein content was higher in vines infested with five and 15 insects compared with non-infested controls (Fig. 1b). Similarly, vines infested with 15 adults had higher protein content 29 days post-infestation when compared with insect-free vines (controls) and with those infested with lower insect densities (5 and 10) (Fig. 1b).

**Fig. 2:**
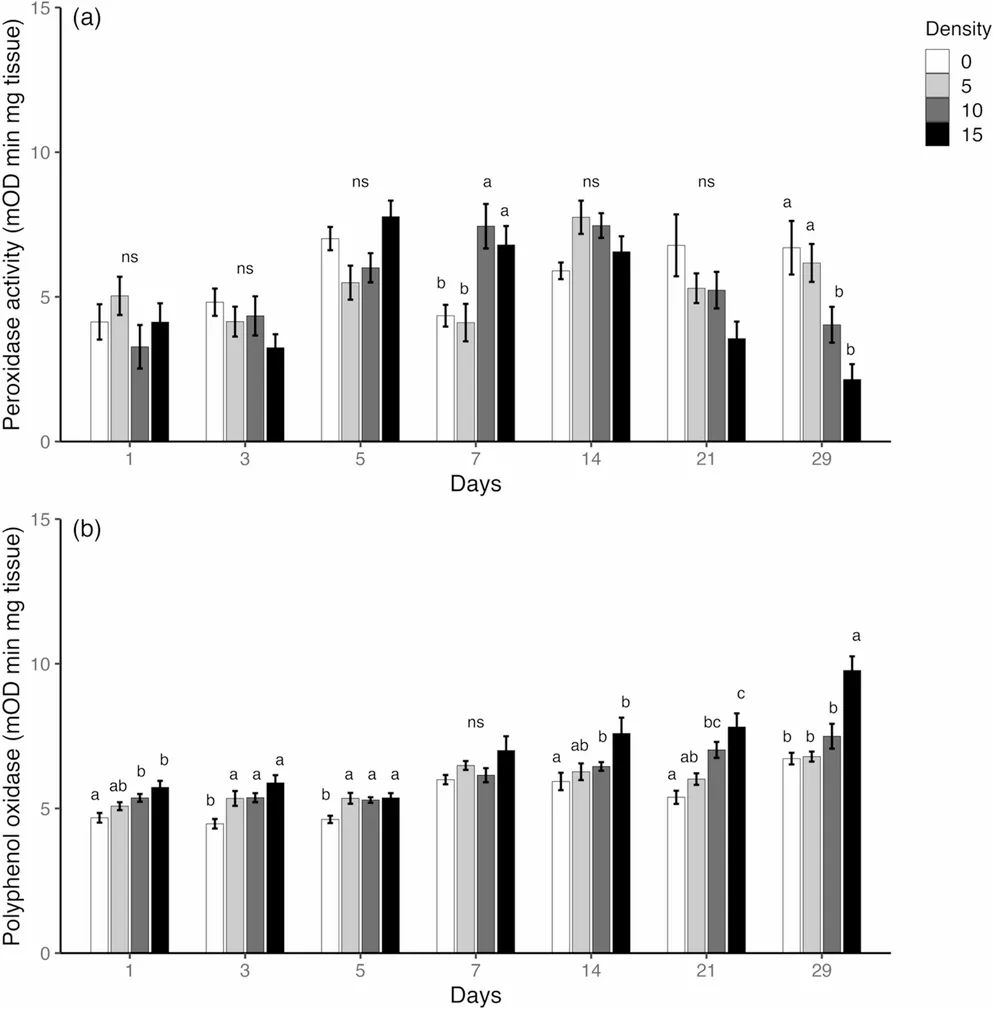
(**a**) Peroxidase and (**b**) polyphenol oxidase activity in grapevine leaves exposed to 0, 5, 10, and 15 spotted lanternfly adults per plant for 1, 3, 5, 7, 14, 21, and 29 days. ANOVA. Letters on bars represent significant differences among treatments (Tukey test at *P* < 0.05) and ‘ns’ denotes non-significance (Tukey test at *P* > 0.05). Bars represent means $\:\pm\:$ standard error

*Spotted Lanternfly Infestation Increased PPO Activity and Induced Changes in the Activity of POX.* The activity of POX varied significantly with insect density (*F* = 2.676, *P* = 0.04) across time points (*F* = 13.599, *P* < 0.0001), with a significant interaction effect (Density x Day: *F* = 4.93, *P* < 0.0001). Significant differences in POX activity were identified at 7 (*F* = 9.119, *P*-adj = 0.00164) and 29 days (*F* = 18.076, *P*-adj < 0.0001) post-infestation, but not at other time points with one-way ANOVA. Subsequent pairwise analyses showed contrasting effects of insect density at these time points. At 7 days post-infestation, vines infested with 10 and 15 insects had higher POX activity than those infested with 5 insects or non-infested controls; however, at 29 days post-infestation, POX activity was lower in vines infested with 10 and 15 insects compared with those infested with zero (controls) and five insects per plant (Fig. 2a).

PPO activity varied among spotted lanternfly density treatments (*F* = 44.553, *P* < 0.0001) and feeding duration (*F* = 49.389, *P* < 0.0001), with a significant interaction between insect density and days of infestation (*F* = 3.118, *P* < 0.0001). One-way ANOVA showed significant effects of insect density at 1, 3, 5, 14, 21, and 29 days post-infestation (Day 1: *F* = 6.877, *P*-adj = 0.0095; Day 3: *F* = 7.135, *P* -adj = 0.008; Day 5: *F* = 9.648, *P*-adj = 0.001; Day 14: *F* = 5.796, *P*-adj = 0.026; Day 21: *F* = 10.437, *P*-adj = 0.00056; Day 29: *F* = 21.622, *P*-adj = 0.026), whereas no significant effect was detected at day 7 (*F* = 2.809, *P*-adj = 0.41; Fig. 2b). PPO activity remained significantly higher in leaves from plants infested with 15 insects when compared with the controls through day 29, except on day 7. Plants infested with 10 insects also had higher PPO activity than the controls at days 1, 3, 5, 14, and 21, whereas plants infested with 5 insects had higher PPO activity than the controls only at days 3 and 5 post-infestation (Fig. 2b).

**Fig. 3:**
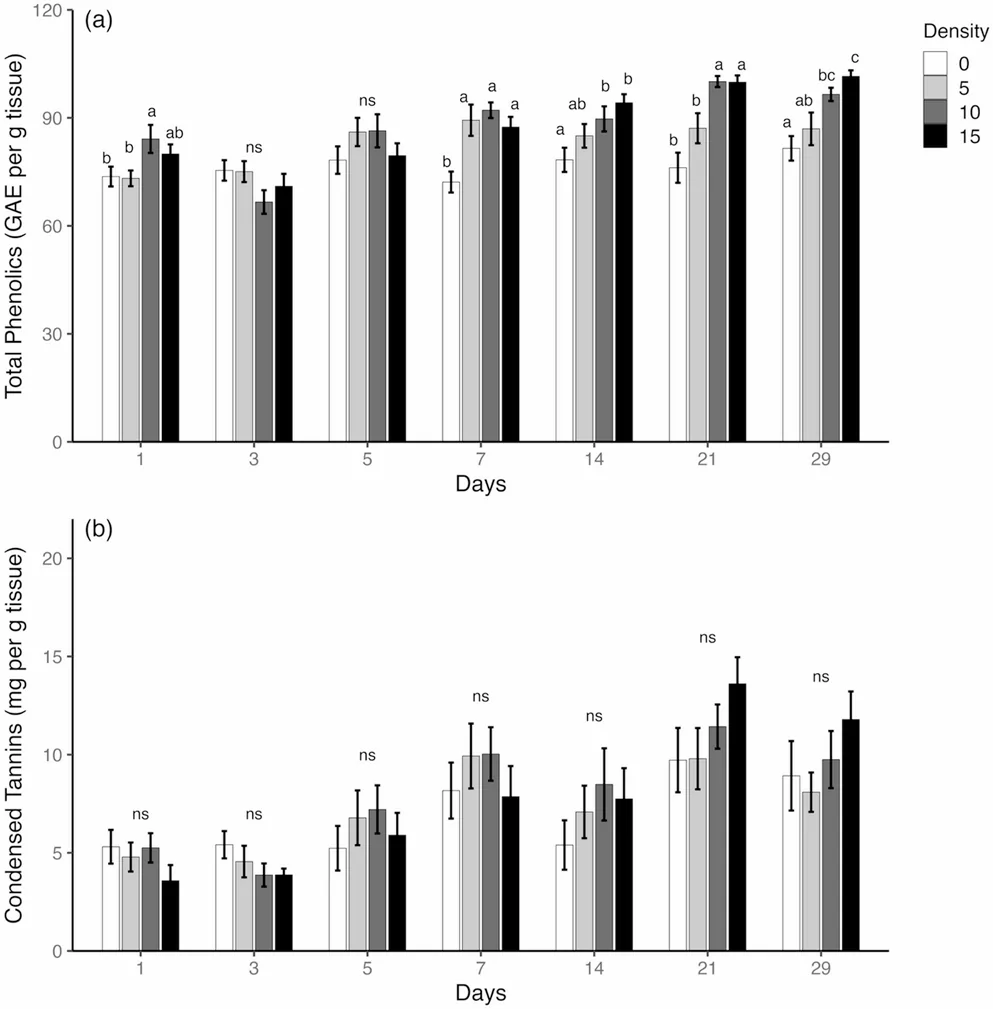
(**a**) Total phenolics and (**b**) condensed tannins in grapevine leaves exposed to 0, 5, 10, and 15 spotted lanternfly adults per plant for 1, 3, 5, 7, 14, 21, and 29 days. ANOVA. Letters on bars represent significant differences among treatments (Tukey test at *P* < 0.05) and ‘ns’ denotes non-significance (Tukey test at *P* > 0.05). Bars represent means $\:\pm\:$ standard error

*Spotted Lanternfly Infestation Increased the Concentration of Total Phenolics but did not Affect the Concentration of Tannins.* The total phenolic content varied significantly with insect density (*F* = 18.856, *P* < 0.0001), feeding duration (*F* = 21.187, p-value < 0.0001), and the interaction of both factors (*F* = 3.622, *P* < 0.0001). One-way ANOVAs indicated that total phenolics accumulation in grapevine leaves changed significantly in response to spotted lanternfly feeding at 1, 7, 14, 21, and 29 days post-infestation (Day 1: *F* = 6.763, *P*-adj = 0.0075; Day 7: *F* = 11.973, *P*-adj = 0.0002; Day 14: *F* = 5.235, *P*-adj = 0.04; Day 21: *F* = 13.02, *P*-adj = 0.0001; Day 29: *F* = 9.32, *P*-adj = 0.002), but not at other time points. Multiple comparisons suggested that at day 1, vines infested with 10 insects had higher phenolic content than those infested with zero (controls) and five insects, with no differences observed among the remaining densities. At 7 days post infestation, the total phenolic accumulation was significantly greater in vines exposed to 5, 10, and 15 insects when compared with non-infested controls. At 14 days, the phenolic accumulation was higher in plants infested with 10 and 15 insects compared with the controls. At 21 days post-infestation, phenolic levels were higher in plants exposed to 10 and 15 insects compared with those infested with 5 or zero (controls). By 29 days post-infestation, total phenolics accumulation was highest in plants infested with 15 insects compared with those infested with zero (controls) and 5; likewise, plants infested with 10 insects also had higher levels than the controls (Fig. 3a).

The concentration of condensed tannins was not influenced by spotted lanternfly density (*F* = 1.577, *P* = 0.195) but was affected by feeding duration (*F* = 18.22, *P* < 0.001); the interaction term was not significant (*F* = 1.316, *P* = 0.178). Subsequent pairwise analysis on feeding times showed an increase in tannin accumulation from 7 to 29 days of feeding, being higher at 21 days (Fig. 3b; Supplementary Fig. 2).

**Fig. 4:**
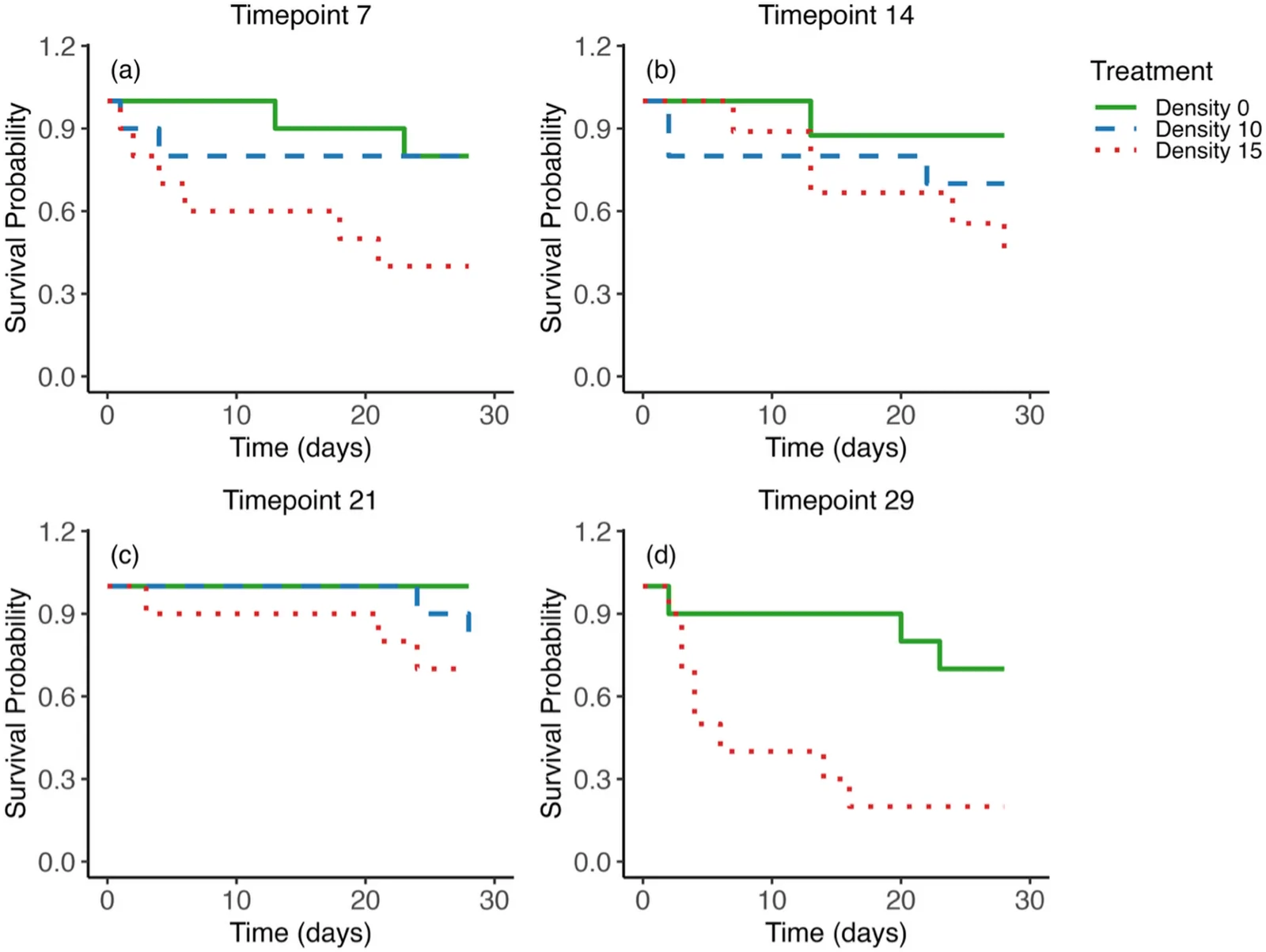
Kaplan-Meier survival curves of spotted lanternfly adults fed on grapevines previously infested with 0, 10 and 15 spotted lanternfly adults for (**a**) 7 days (timepoint 7, *P* = 0.07); (**b**) 14 days (timepoint 14, *P* = 0.2); (**c**) 21 days (timepoint 21, *P* = 0.2), and (**d**) 29 days (timepoint 29, *P* = 0.01) post-infestation

**Fig. 5:**
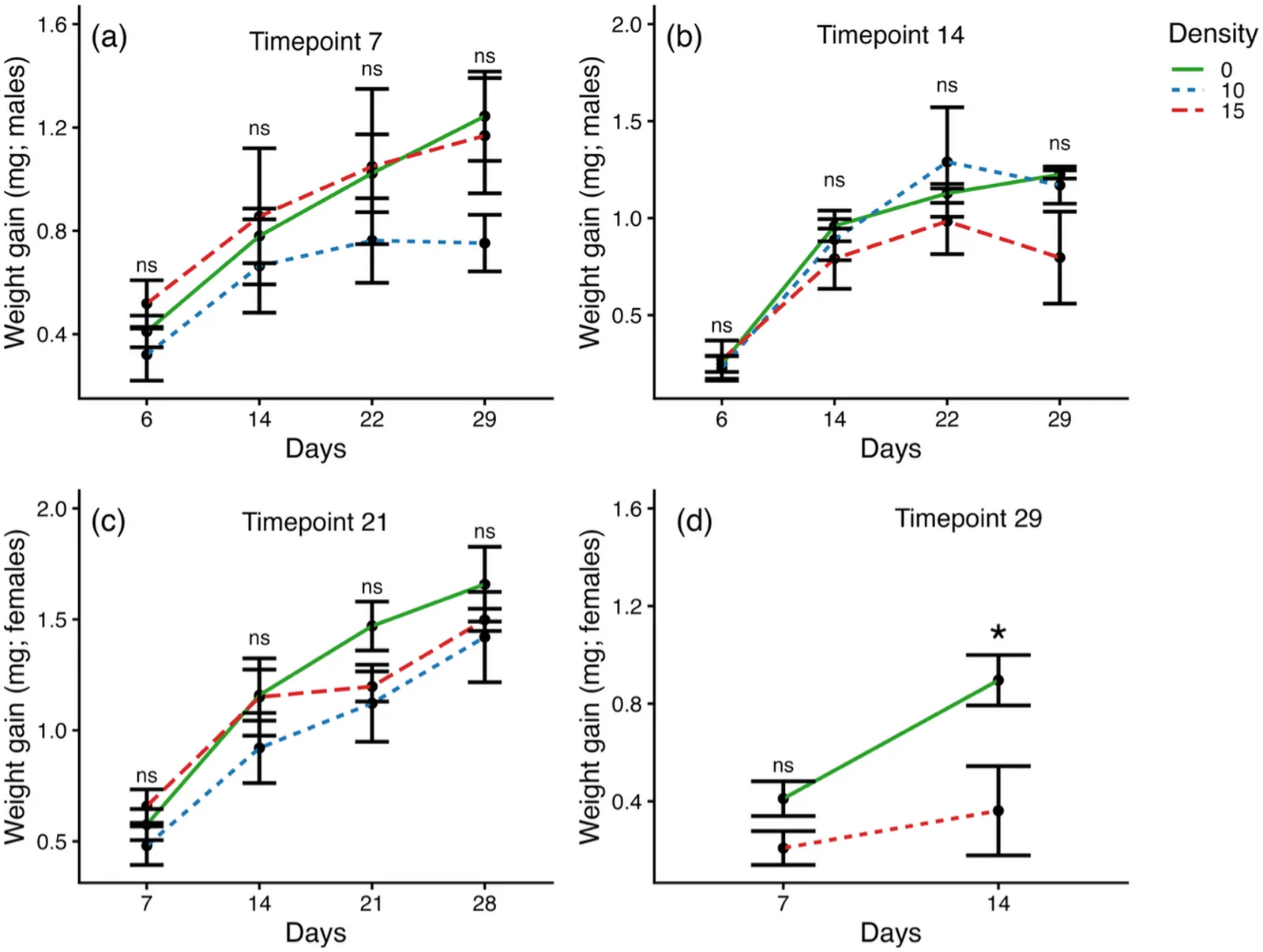
Weight gain of spotted lanternfly adults over time when fed on grapevines previously infested with 0, 10, and 15 spotted lanternfly adults for (**a**) 7 days; (**b**) 14 days; (**c**) 21 days; and (**d**) 29 days. Linear Mixed-Effects Model. Asterisks (*) on error bars show significant differences between treatments at a given timepoint (Tukey test at *P* < 0.05), and ‘ns’ denotes non-significance (Tukey test at *P* > 0.05). Black dots with error bars represent means $\:\pm\:$ standard errors

*Prolonged Spotted Lanternfly Infestation Reduced Subsequent Spotted Lanternfly Fitness.* We evaluated the survival and weight gain of newly emerged spotted lanternfly adults fed on grapevines that had previously been infested with different spotted lanternfly densities (0, 10, and 15 insects per plant) for different time periods (7, 14, 21, and 29 days). We did not find statistical differences in the survival of young adults when fed on grapevines previously exposed to adult lanternflies for 7, 14, and 21 days compared with those fed on non-insect-infested plants (Kaplan-Meier; *P* > 0.05, Fig. 4a-c). However, the survival of freshly emerged adults was significantly reduced when fed on grapevines previously infested with 15 adult lanternflies for 29 days compared with those fed on non-infested control plants (Kaplan-Meier; *P* = 0.01, Fig. 4d). Similarly, newly emerged adults fed on grapevines previously exposed to adult feeding for 7 (LMM; Density: *F* = 1.199, *P* = 0.33; time during bioassay: *F* = 37.959, *P* < 0.0001; Density x time during bioassay: *F* = 1.418, *P* = 0.23), 14 (LMM; Density: *F* = 1.136, *P* = 0.35; time during bioassay: *F* = 39.31, *P* < 0.0001; Density x time during bioassay: *F* = 1.324, *P* = 0.28), and 21 (LMM; Density: *F* = 1.22, *P* = 0.32; time during bioassay: *F* = 48.1, *P* < 0.0001; Density x time during bioassay: *F* = 0.96, *P* = 0.46) days, gained similar weight on infested and non-infested control plants (Fig. 5a-c). But their weight gain was significantly reduced when fed for two weeks (14 days) on grapevines previously exposed to 15 lanternfly adults for 29 days (LMM; Density: *F* = 10.37, *P* = 0.007; time during bioassay: *F* = 10.85, *P* = 0.007; Density x time during bioassay: *F* = 3.8, *P* = 0.07, Fig. 5d).

## Discussion

In agreement with our hypothesis, this study reveals that infestation by 15 spotted lanternfly adults per plant reduced chlorophyll content in grapevine leaves and induced systemic defense responses that reduced insect survival and weight gain after four-weeks of infestation. Key defense-related enzymes and compounds were induced in response to spotted lanternfly feeding, including PPO, POX, and total phenolics, though POX activity was reduced with prolonged feeding. Furthermore, bioassays conducted with newly emerged adults showed higher mortality and lower weight gain in insects fed on previously infested grapevines compared with those fed on controls. Together, these findings suggest grapevine defense activation under high spotted lanternfly feeding pressure that may affect plant growth and reduce insect survival.

Chlorophyll content was reduced in response to high-density spotted lanternfly feeding after 21 days. Chlorophyll functions as the primary light-absorbing pigment in plants, capturing solar energy for photochemical conversion during photosynthesis (Mandal and Dutta 2020). Because spotted lanternflies remove large quantities of phloem sap during prolonged feeding, they may alter source-sink relationships, reduce carbon transport to sink tissues, and induce water stress, all of which can reduce photosynthetic function. Reductions in chlorophyll may therefore contribute to lower photosynthesis rates, which can ultimately impair plant growth and reduce yield. Consistent with this interpretation, reductions in carbon assimilation and photosynthesis have been previously reported in grapevines (*V. vinifera* cultivar Riesling) infested with spotted lanternflies (Harner et al. 2022). The spotted lanternfly has also been reported to reduce photosynthetic rate and stomatal conductance when feeding on red (*Acer rubrum*) and silver (*A. saccharinum*) maples (Lavely et al. 2022). Furthermore, spotted lanternfly feeding reduces root biomass and total non-structural carbohydrates in 3-year-old grapevines (*V. vinifera* cultivar Cabernet Franc) (Harner et al. 2025), suggesting disruption of plant carbon allocation. In general, sap-feeding insects typically suppress plant growth and photosynthesis as indicated in a meta-analysis conducted by Zvereva et al. (2010). Consequently, the reduction in chlorophyll suggests that spotted lanternfly feeding may be impairing photosynthetic capacity, which could negatively affect grapevine growth.

Spotted lanternfly feeding induced higher activity of JA- and SA-regulated enzymes. We found consistent induction of PPO activity after one day of feeding through day 29 (except on day 7). PPO is commonly induced by herbivory and contributes to plant resistance against insect herbivores (Duffey and Felton 1991; Felton and Duffey 1992; Duffey and Stout 1996; Felton 2005). PPO oxidizes phenolic compounds to quinones that bind to plant proteins and reduce their digestibility in the insect gut. PPO can also lead to the formation of melanin through the oxidation and polymerization of phenolic compounds, which can reduce nutrient availability, decrease protein digestibility, and lead to oxidative stress in insect herbivores (War et al. 2012). In grapevines, PPO induction has been reported in response to both pathogen infection and arthropod herbivory (Rusjan et al. 2012; Shi et al. 2022; Sree et al. 2024). Shi et al. (2022) reported increased PPO activity in *V. vinifera* (cultivar Cabernet Sauvignon) leaves following feeding by the mite, *Colomerus vitis* (Prostigmata: Eriophyidae). Elevated PPO activity has also been documented in grapevines infected with powdery mildew (*Erysiphe necator*) and Bois noir disease (Rusjan et al. 2012; Sree et al. 2024). Additionally, Islam et al. (2022) reported the upregulation of two polyphenol oxidase genes in grapevines in response to spotted lanternfly feeding. Induced PPO activity in grapevines may increase oxidative stress, reduce spotted lanternfly nutrient uptake, and lower the insects’ digestive efficiency, providing some degree of resistance to this insect.

POX activity was induced by spotted lanternfly feeding 7 days after infestation, but it was later suppressed (by day 29) at higher insect densities (10 and 15 adults per vine). These results contrast with those from a previous study that identified 13 (out of 19) peroxidase genes upregulated in phloem tissue from grapevines (hybrid Marquette) infested with 80 spotted lanternfly adults for 42 days (Islam et al. 2022). These discrepancies may be attributed to differences in cultivar responses to lanternfly feeding and variations in experimental conditions between the two studies. POXs contribute to the formation of lignin, which strengthens cell walls, contributing to enhanced resistance against pathogens and insect herbivores (Dai et al. 1995; War et al. 2012; Liu et al. 2018; Perez-Alonso et al. 2025). An additional important role of POXs is the regulation of ROS accumulation (Do et al. 2003; Schaffer and Bronnikova 2012) induced by pathogen and herbivore damage (Gambino et al. 2013; Suman et al. 2021; Sahu et al. 2022). Although oxidative bursts provide a rapid response that activates downstream defense pathways and reduces tissue palatability and digestibility to insects, the excessive accumulation of ROS can be harmful for plants; therefore, oxidative enzymes are critical to maintain ROS balance (Torres 2010; Sharma et al. 2012). The role of POXs in response to insect feeding has been understudied in grapevines, but studies with mites (*C. vitis*) found reduced activity in *V. vinifera* (cultivar Cabernet sauvignon) (Shi et al. 2022), and increased activity in other cultivars (Javadi Khederi et al. 2018), suggesting cultivar-dependent responses. Nevertheless, grapevines upregulate POXs in response to pathogen infection, such as *Plasmopara viticola* (downy mildew) and grapevine leafroll-associated virus, confirming their importance as a defense mechanism (Dai et al. 1995; Harm et al. 2011; Bayramova et al. 2021). In our experiments, prolonged spotted lanternfly feeding reduced POX activity, possibly through the action of salivary effector proteins. Because POXs are also involved in the lignification of cell walls, POX suppression by spotted lanternflies may facilitate stylet penetration into plant tissues in addition to reducing oxidative damage at the feeding sites.

Leaf protein content was increased after 7 and 29 days of high-density spotted lanternfly feeding, suggesting that plants might be activating and accumulating protein-based defense responses, which can be supported by the increase in activity of defensive enzymes (PPO). Previous studies have reported increases in leaf protein content elicited by insect herbivory in other plant-herbivore systems (Chen et al. 2009; War et al. 2013).

Total phenolics were consistently accumulated in grapevines infested with 10–15 lanternflies for 7–29 days. These results confirm those reported by Islam et al. (2022), who found several upregulated genes in the phenylpropanoid pathway encoding key enzymes in phenolic biosynthesis (e.g., phenylalanine ammonia-lyase, flavonoid 3’-monooxygenases, flavonoid-3’-hydroxylase, etc.) following spotted lanternfly feeding in Marquette grapevines. The accumulation of phenolic compounds has been documented in grapevines in response to both pathogen infection and arthropod herbivory (Rusjan et al. 2012; Javadi Khederi et al. 2018; Eitle et al. 2019; Sree et al. 2024). For example, grape phylloxera *Daktulosphaira vitifoliae* (Hemiptera: Phylloxeridae) infestation induces flavonoid and stilbene accumulation in grapevine roots (Eitle et al. 2019). In this study, increased phenolic concentrations coincided with elevated PPO activity and reduced spotted lanternfly survival and weight gain, suggesting a coordinated biochemical defense response that may contribute to decreased host suitability. However, because phenolic biosynthesis is metabolically costly, sustained accumulation may also reflect a potential growth-defense trade-off, where resources are allocated toward defense at the expense of growth-related processes. Plant phenolics can either be toxic to insect herbivores by causing oxidative damage in their midgut or increase feeding deterrence due to reduced tissue palatability; through these mechanisms, phenolics can affect insect growth, development, and reproduction (Duffey and Isman 1981; Duffey and Stout 1996; Singh et al. 2021). This suggests that the accumulation of phenolics may contribute to grapevine resistance to spotted lanternfly.

Grapevine responses to spotted lanternfly feeding may result from mechanical injury inflicted by the insect’s mouthparts, the plant recognition of molecules secreted in the insect’s saliva, and the water and nutrient loss induced by extensive sap removal from the insect. The spotted lanternfly salivary glands contain effector proteins and phytohormones that, upon secretion, may modulate the induction of plant responses (Acevedo 2024; Smith et al. 2026). For instance, spotted lanternfly contains high concentrations of SA in its salivary glands that, if secreted, may induce pathogen-associated defense responses, reducing JA-associated herbivore defenses due to hormone crosstalk, allowing the insect to thrive. However, the upregulation of auxins may antagonistically interfere with SA, allowing JA levels to increase. Additionally, extensive spotted lanternfly feeding seems to trigger the induction of stress-related hormones such as ABA. These results highlight a complex regulation of grapevine defense responses mediated by several phytohormones.

Our bioassays showed a reduction in spotted lanternfly survival and weight gain when feeding on grapevines previously exposed to 15 insects for 29 days. This suggests that prolonged, high-density feeding reduces spotted lanternfly fitness either through enhanced plant defenses, a reduction in plant nutrient availability, or the combination of both mechanisms. This study demonstrates induction of defense responses (increase in PPO activity and phenolic concentrations), but nutrient analyses were not conducted. Prolonged feeding by sap-sucking insects can also reduce the nutritional quality of plant sap by diverting photoassimilates toward the feeding site and depleting plant resources (Zhou et al. 2015). Studies on aphids *Macrosiphoniella tanacetaria* and *Uroleucon tanaceti* (Hemiptera: Aphididae) and *N. lugens* found that their feeding alters phloem sugar and amino acid composition in tansy (*Tanacetum* vulgare) and rice (*Oryza sativa*), respectively, creating localized nutrient sinks that enhance insect nutrition (Jakobs et al. 2019; Yu et al. 2024). Sap-sucking insects can also disrupt sucrose and water transport in the plant, further contributing to a decline in photosynthesis (Douglas 2006). Long-term spotted lanternfly feeding appears to be detrimental to plant health by depleting photo assimilates, disrupting carbon transport, inducing oxidative stress, and changing water and nutrient balance, which can reduce yield (Harner et al. 2022).

In conclusion, our findings indicate that spotted lanternfly feeding induces systemic grapevine defense responses that are stronger at higher insect densities and are expressed in a time-dependent manner. PPO activity and the accumulation of phenolics were induced within the first week of insect infestation and remained high in insect-infested plants throughout the experiment, but reductions in chlorophyll content were not found until after three weeks of feeding by 10–15 insects per plant. These results suggest that grapevine physiological responses increase with infestation intensity and feeding duration. Furthermore, the induced responses were associated with lower survival and reduced weight gain of lanternflies subsequently fed on previously infested plants, indicating that prolonged feeding decreases host suitability, reduces insect fitness, and may contribute to induced resistance. Collectively, our results provide new insights into the mechanisms underlying grapevine responses to spotted lanternfly infestation. Further research is needed to identify insect-derived elicitors or effectors and plant signaling mechanisms involved in these responses and determine whether these responses differ among grapevine cultivars.

## Supplementary Information

Below is the link to the electronic supplementary material.

Supplementary Material 1

Supplementary Material 2

## Data Availability

Data available upon request.
