# Supplementary material for: Spotted Lanternfly Feeding Induces Systemic Defense Responses in Grapevines
Source: J Chem Ecol. 2026 Jul 24;52(4):67. doi: 10.1007/s10886-026-01742-2 (PMC13400602; doi:10.1007/s10886-026-01742-2)

**Spotted lanternfly feeding induces systemic defense responses in grapevines**

^1^Sukhman Singh, ^1^Michelle Peiffer, ^1^Gary W. Felton and ^1^Flor E. Acevedo

Department of Entomology, The Pennsylvania State University, University Park, 16802, PA, United States

Corresponding author: Flor E. Acevedo, [fea5007@psu.edu](mailto:fea5007@psu.edu)

**Supplementary figure 1**: (a) Chlorophyll A and (b) Chlorophyll B in grapevine leaves fed on by ‘0’, ‘5’, 10’, and ‘15’ spotted lanternflies per plant over time (1, 3, 5, 7, 14, 21, and 29 days). ANOVA. Letters on bars represent significant differences among treatments (Tukey test, P<0.05) and ‘ns’ denotes non-significance (Tukey test, P>0.05). Bars represent means ± standard error of the means.


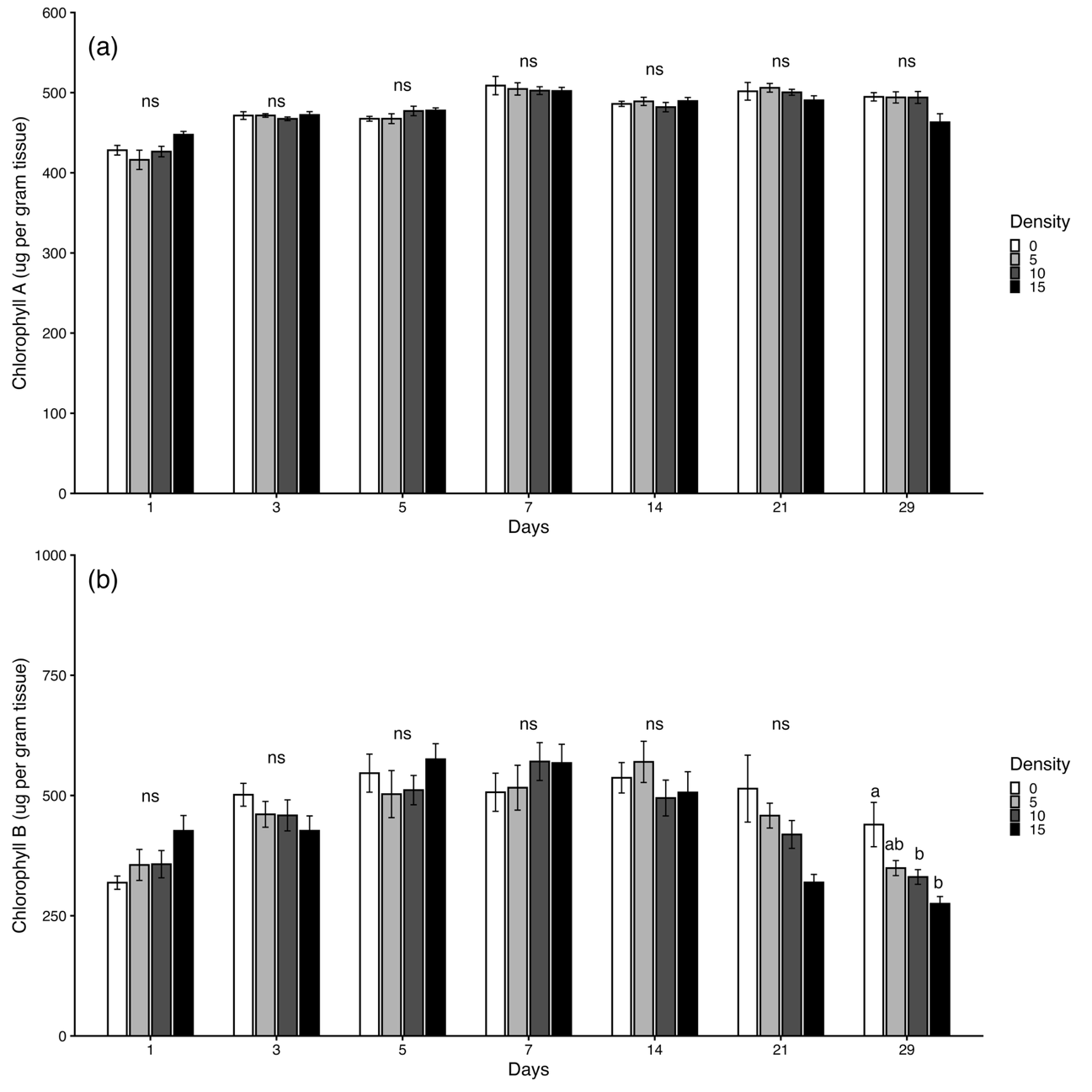


**Supplementary figure 2**: Condensed tannin content in grapevine leaves over time (1, 3, 5, 7, 14, 21, and 29 days) regardless of insect density treatment. Letters on bars represent significant differences among treatments (Tukey test, P<0.05). Dots represent means ± standard error of the means.


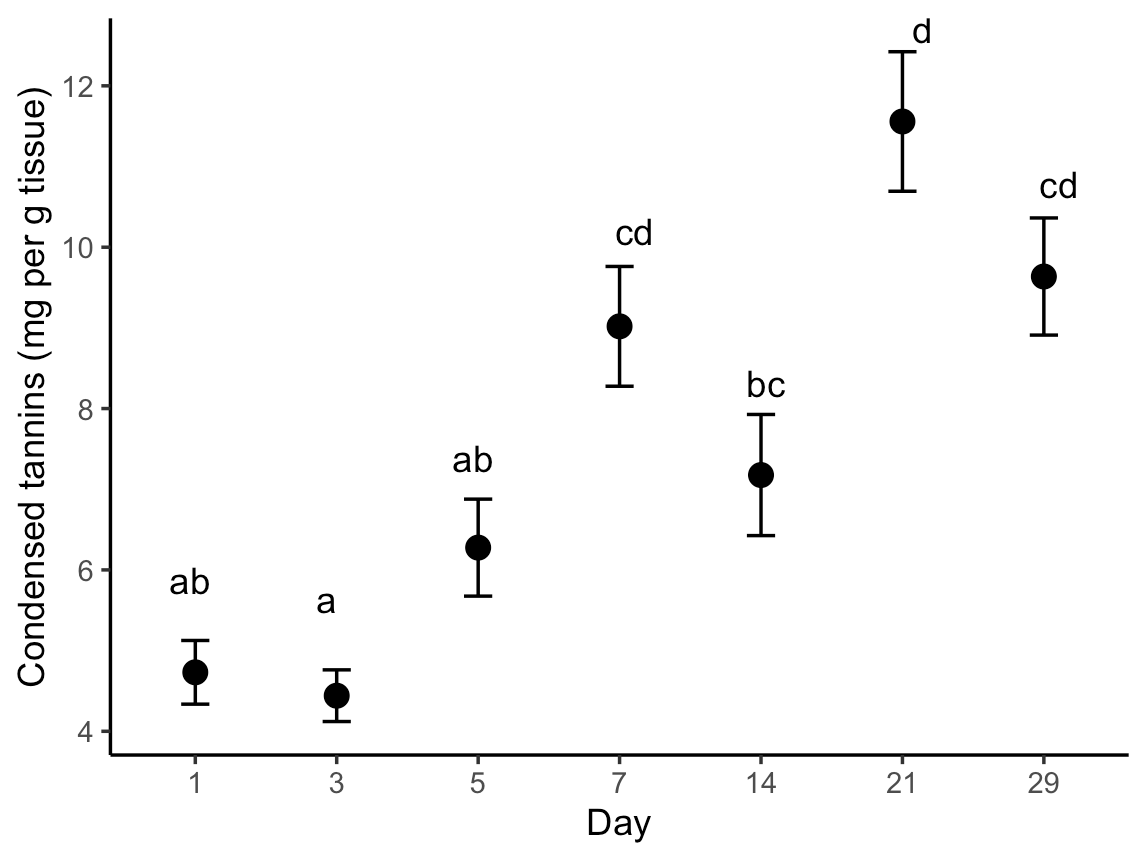

Supplement: Supplementary file 2 — Supplementary Material 2 [file 10886_2026_1742_MOESM2_ESM.docx]
